# Gems

**Published:** 1883-01

**Authors:** 


### Gems.

The following remarks by the editor of the *Western Medical Reporter* are timely, and especially so, if medical men will be by such cautions induced to accept the various theories that are advanced with due care and allowance.

Some of the most plausible theories, that have been advanced within fifteen years past; and sometimes with much assurance of correctness, and based upon large and thorough experiment; have required reconsideration, revision, and sometimes an entire replacement. Investigation is of inestimable value, and new ascertainments can not be too highly appreciated, and especially after having been fully verified. EDITOR REGISTER.

